# Remapping cybersecurity competences in a small nation state

**DOI:** 10.1016/j.heliyon.2023.e12808

**Published:** 2023-01-10

**Authors:** Linas Bukauskas, Agnė Brilingaitė, Aušrius Juozapavičius, Daiva Lepaitė, Kęstutis Ikamas, Rimantė Andrijauskaitė

**Affiliations:** a Institute of Computer Science, Vilnius University, Didlaukio Str. 47, LT-08303 Vilnius, Lithuania; b General Jonas Žemaitis Military Academy of Lithuania, Šilo Str. 5A, LT-10322 Vilnius, Lithuania; c Institute of Educational Sciences, Vilnius University, Universiteto Str. 9, LT-01131 Vilnius, Lithuania

**Keywords:** Cybersecurity competences, Workforce capacity, IT security, Activity-based competence map

## Abstract

The impact of cybersecurity (CS) on public well-being is increasing due to the continued digitisation process of all industry sectors. The protection of information systems rests upon a sufficient number of CS specialists and their competences. A cyber-competence map describing the capacity and trends of the CS workforce is an essential element of the workforce development strategy. Large enterprises tend to have narrowly specialised employees with clearly identifiable roles. Still, most enterprises in small countries are SMEs. Therefore, the tasks and responsibilities of many CS-related specialists overlap the functions of several roles. This paper aims to develop a small-state cybersecurity competence map consistent with the standards of professional organisations. The work applies a combined qualitative and quantitative methodological approach to collect data using questionnaires and expert interviews in the CS field organisations. The study includes a representative public survey, a large-scale survey of company executives, an exploratory CS expert survey, and a comprehensive job posting analysis. Finally, a national CS competence map is presented and verified using two qualitative semi-structured interviews with field professionals. Even though the map reflects a status of a small nation state, it is activity-based and might be applicable in any country. As a future research direction, we will investigate the impact of early and late exposure to cybersecurity competences in education and framework applicability.

## 1 Introduction

The rapid advancement of technologies and transfer of services to cyberspace inevitably leads to an increased number of cyber incidents that threaten national economic and political stability [30]. Global supply chains are under threat as cybercrime impacts private individuals and causes major disruptions, significant financial losses, and reputation damage to many enterprises and organisations. Cybersecurity (CS) specialists tasked with the prevention of these incidents usually have Information Technology (IT) or Information Communication Technology (ICT) education background, but the problem should be addressed at all levels. Strategic cybersecurity management is essential in minimising data breach risk [1].

A constant drive for time efficiency forces many business sectors to experience digital transformation in services and workplaces by applying state-of-the-art ICT tools. Therefore, their resilience against incidents and disruptions depends on a combination of transformative capacity and cybersecurity [16]. Innovative solutions are required to process and analyse data, protecting users' privacy, for example, in the health sector [24]. The IT sector is actively involved in developing tools for the digital society, and manufacturers aiming to become innovative solution providers have to move the focus from the product logic to the service logic [19]. Stakeholders envision resilient systems and infrastructure as a common goal; therefore, challenges in security-relevant sectors are indicative of CS trends and provide directions for future research and innovation [13].

The higher education sector is reacting to the increased demand for CS specialists with a plethora of CS study programs worldwide. Moreover, sectorial communities are contributing by developing the generalised multipurpose frameworks of cybersecurity skills—one of the best known is the NIST NICE framework [31]. The frameworks provide the rationale of profiles, alternative job titles, tasks performed by the profile, key knowledge, and essential skills. Still, the competences of CS specialists need to be explicitly defined and mapped to tasks. An explicit definition of needed competences for different levels of roles could support the mapping exercise. It would justify educational routes to design qualification degrees or propose lifelong learning curricula in the CS sector.

Demand for CS specialists grows in Lithuania the same way as in other countries [14], even if the country has a high rank according to the global cybersecurity index. However, it is challenging to meet the demand when the CS sector requires specific technological knowledge from several overlapping areas and general competences. Therefore, it is necessary to have a national-level recruitment strategy and coordinated education of the CS specialists to meet future workforce demands. The experience of other countries shows a need to attract non-technological specialists to the area.

The work aims to develop a national cybersecurity competence map to support a security-oriented ecosystem and foster innovation development in the digital society. While creating a cybersecurity framework, it is important to analyse the alignment of the current frameworks with the *status-quo* of the cybersecurity workforce in a small nation state.

We formulate our hypothesis in the context of the aim: *Current cybersecurity skill and competence frameworks do not represent the workforce profiles in a small nation state's labour market.* We designed a multi-phase research methodology workflow and included quantitative and qualitative components to triangulate results. Questionnaires, analysis of job postings, and interviews with multiple data-gathering points enabled the testing of the hypothesis. The collected data enabled us to propose an alternative competence framework. We contribute to the CS community with a hierarchical competence framework that balances the workforce proportions for educational and business purposes.

The paper is structured as follows. Section 2 presents the background of the work with a literature overview. Section 3 defines the methodology for the research setup and analysis workflow, with results presented in Section 4. Discussion of the results leads to a proposed competence framework in Section 5. The work concludes in Section 6 with possible future research directions.

## 2 Background

Globally, cybersecurity is treated as a branch of computer science, even though principles originating from other research and study fields, such as management and law, constitute a mandatory part of some specific CS-related topics. Cybersecurity Curricula 2017, CS2017, by professional computer science communities [20], is one of the standards to follow when developing or updating a study program associated with CS. It defines CS discipline and divides the CS-related content and topics into several knowledge areas. For example, *Risk management*, *Governance and policy*, *Laws, ethics, and compliance*, and *Strategy and planning* are essentials of the knowledge unit of *Organisational security* to cover mostly non-IT topics. The knowledge unit of *System security* includes *Holistic approach*, *Security policy*, *Authentication*, *Access control*, *Monitoring*, *Recovery*, *Testing*, and *Documentation*. This unit combines the abilities to correlate policies and technical implementations of system security.

CS2017 follows the professional NIST NICE competence framework [29], [31]. NIST NICE describes tasks and associates them with skills and knowledge required to perform a work role. The framework contains several roles described in detail, falling into several categories: *Securely Provision*, *Operate and Maintain*, *Oversee and Govern*, *Protect and Defend*, *Analyze*, *Collect and Operate*, and *Investigate*. One category covers several dedicated roles with different work scopes. For example, the category *Securely Provision* includes Risk Management (e.g. authorising official), Software development (e.g. software developer), Systems Architecture (e.g. enterprise architect), and others.

NIST NICE model distinguishes small scope-oriented roles, and the framework's applicability might be limited due to the lack of the workforce in smaller enterprises of small nation states. The European Cybersecurity Skills Framework, ECSF [11], contains 12 cybersecurity roles providing a more general view of the CS workforce than NIST NICE: 1) chief information security officer (CISO), 2) incident responder, 3) legal, policy and compliance officer, 4) threat intelligence specialist, 5) architect, 6) auditor, 7) educator, 8) implementer, 9) researcher, 10) risk manager, 11) digital forensics investigator and 12) penetration tester. The implementer role is an umbrella for all cybersecurity implementation-related aspects, including infrastructure solutions and products (systems, software, services).

European regulations and national legislation define several specific roles in CS. For example, General Data Protection Regulation, GDPR [37], enforced the introduction of the data protection officer position required for the public sector and some businesses. Also, the qualification frameworks define the levels based on skills, knowledge, responsibility and autonomy level in a work position as a standard. The European Qualification Framework [35] defines qualification levels to transfer between national qualification frameworks. For example, Level 6 requires demonstrating mastery and innovation to solve complex and unpredictable problems, and Level 7 includes reviewing the strategic performance of teams.

Cybersecurity is not listed as a separate field in the study field classifier in Lithuania. National higher education institutions design and improve study programs following study field descriptions [5]. When searching for keywords related to cybersecurity (e.g. “cyber security,” “cyber incidents,” “cyberspace,” and “electronic information security”), the results appear only in the study field group of computing [4]. The rest of the descriptions of the study fields did not contain any of the keywords. Therefore, competences related to cybersecurity overlap with computer science. In the description of the computer science field, it is stated that *“The core of the group of study fields of computing consists of the following areas of knowledge: (...) security of information and information technologies, including the aspects of cyber security (...)”*.

European Skills, Competences, Qualifications and Occupations (ESCO) classification [36] defines qualifications for the European labour market and education. Compared to the above-listed frameworks, ESCO assumes that a security architect, security advisor, and security consultant are alternative names for an ICT security engineer. Still, they are separate roles in the ENISA ECSF model and, of course, in the detailed NIST NICE model.

The competence frameworks and qualification classification differ in their level of detail and might be ill-suited to describe existing CS roles in smaller countries. Therefore, applying these frameworks could negatively impact strategic workforce development. Consequently, there is a need to create a national CS competence map adapted to the reality of a small country to ensure that all stakeholders use the same CS vocabulary.

## 3 Methodology

**Figure 1:**
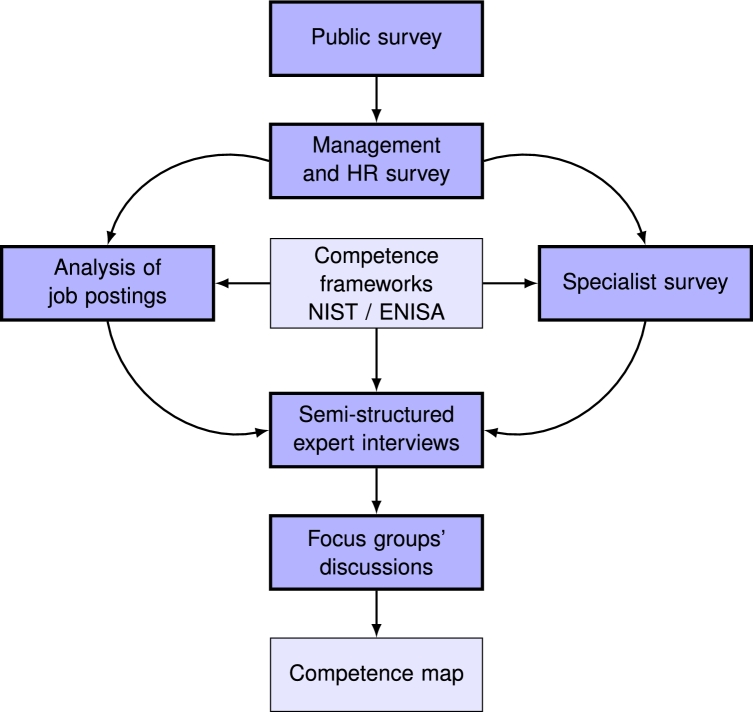
Research methodology.

We conducted a multi-level qualitative and quantitative study of cybersecurity roles in Lithuania. Fig. 1 presents the overall view of the methodology used to test our hypothesis.

### 3.1 Research steps

To understand the broader picture, we started with a quantitative representative public survey, followed by a survey of chief executives (CEO), including human resources managers (HRM) (see Fig. 1). Then, to see the current application of two major competence frameworks (NIST NICE and ENISA ECSF) in the country's labour market, we collected and analysed cybersecurity-related job postings and performed an exploratory survey of CS specialists. To extract further details about the requirements for CS specialists, we conducted a set of semi-structured interviews with experts in the area and organised two focus group discussions. The findings support our research hypothesis, and therefore we propose a CS competence map suitable for a small country.

Data collection in the quantitative part (surveys) is carried out using questionnaires, whereas qualitative content analysis [8] with an induction approach is applied when dealing with expert interviews. Methodological triangulation of different methods to confirm findings ensured the study's validity. All interview participants were informed about the study's objectives and agreed to participate. According to GDRP regulation [37] and national legislation, the data is kept anonymous, with any private information removed.

Interviews with participants were conducted according to the established ethical guidelines of the Code of Academic Ethics and Regulations of the Academic Ethics Commission of the Core Academic Units of Vilnius University. In compliance with Order, No. V-60 of the Ombudsperson for Academic Ethics and Procedures of the Republic of Lithuania, 2020 Section IV, paragraph 27, interviews qualified for an exemption of ethical review board approval. The participant group did not include vulnerable persons, and no intervention methods were applied. All ethical principles were assured, and written consent was received as voluntarily expressed declarations. All participants had the possibility to leave and stop interviews at any time. Gathered data were managed according to the data management plan approved by the Research Council of Lithuania.

### 3.2 Research sample and statistics

Cybersecurity is a relatively new field that arose naturally from the broader ICT field. It would not be surprising if the role of a cybersecurity specialist in the mind of an ordinary society member is therefore confused with the usual system administrator. A representative population survey (Omnibus) was the first step in our research, and we included several questions to identify the general population's understanding of the cybersecurity profession. The Omnibus survey was carried out in Lithuania in September–October 2021. In total, 1004 persons of ages 18 and older were surveyed in every region of the country. They were chosen using a multi-stage statistically random selection process and individual interviews so that the distribution of respondents would closely match the population distribution according to gender, nationality, age, and area of residence. The maximum error of results is 3%, given the sample size. A public opinion polling company implemented the survey using a questionnaire designed by us. Table 1 presents the summary of data sample sizes used in producing the results. Complete questionnaires are provided as supplementary material for the paper.

**Table 1:** Overview of data samples used.

Data set	Data size	Purpose
Omnibus	1004	public view and opinion
CEO & HRM	246	top-down view of CS
Job postings	175	competences needed
CS specialist	29	bottom-up view of CS
Expert interview	5	career paths
Focus groups	2	views of relevant groups

The tasks and responsibilities of CS personnel in an organisation depend on the resources allocated. Therefore the next stage of the research concentrated on the opinion of top managers (CEOs and HRMs) of various public and private organisations. The smallest companies rarely have a dedicated CS or IT specialist, so we intentionally limited their number in the survey. Companies with less than 50 employees constituted only 15% of the respondents. The remaining larger companies matched the size distribution of businesses in the country. In total, 1343 company managers were contacted, 252 completed the questionnaire via a phone call or a web form, and 246 responses were found valid. There were 2820 companies with more than 50 employees in Lithuania at the time, and the sample size of 208 respondents from those companies (85% of the responses) gives us a confidence interval below 7% for the 95% confidence level. The response rate was unusually high due to the relevance of the topic and the reputation of the institutions performing the research. A public opinion polling company was commissioned to carry out the survey according to our requirements.

A small exploratory survey was designed and performed to determine the overlap of CS roles (specified in the two competence frameworks) among the functions of CS specialists in various organisations. The survey questionnaire consisted of job functions used in the NIST NICE framework and areas from ENISA ECSF. We chose 35 CS specialists via their professional public profiles on the LinkedIn professional network and asked them to specify CS tasks carried out in their companies. We received and analysed 29 responses. To further cross-correlate findings, we set up several semi-structured interviews with experts in the field.

Researching online job postings is a way to identify the needs and requirements for a profession or a position in a chosen field. This analysis based on two dominant job posting platforms complements the surveys of CS experts, company executives, and the general public. It provides a more comprehensive picture of the CS sector. Similar joint studies are being carried out in other countries. [18] studied job advertisements, conducted surveys, and then identified sets of competences specific to Australia. Using CS-related keywords, we manually identified and analysed 100% of available CS job postings (175 out of more than 4000 ICT-related postings). Two focus group discussions and five expert interviews supported the results from the quantitative part. The experts were chosen based on their experience in senior work positions and active participation in national cyber defence exercises and the national cybersecurity community.

## 4 Results

### 4.1 Results of the public survey

A representative survey of the population revealed the public opinion about the role of a CS specialist. The respondents were asked to identify the CS function and select the best applicable definition out of four choices:

F1. Monitors activity of computer systems and reacts to security incidents.
F2. Develops websites and information systems.
F3. Manages computer networks and computers.
F4. Develops mobile applications and/or computer games.
F5. Did not know or did not answer.

The respondents were expected to choose the F1 answer as the most likely function of a CS specialist because it had the exact keyword “security” as the role name, and it was the first of the possibilities. Whoever chose a different option was not familiar with the role.

However, only slightly over half of the respondents (57.9%) chose F1 as an answer. The highest percentage of correct answers came from the youngest generation of men (under 25), with 70.5% choosing F1 (see Table 2). In total, more than a third of the population could not identify the functions of a CS specialist, and 12.4% selected the functions of a systems administrator. Therefore, according to public opinion, the role of a cybersecurity specialist is relatively young and unknown, with a tendency to confuse it with the role of a generic IT specialist.

**Table 2:** Primary function of a CS specialist—public opinion of different genders and age groups (F1 is the correct choice, F3 denotes functions of a system administrator, others are incorrect, F5—did not answer).

	Males					Females					Total
Answer Age	≤ 25	26-45	46-65	> 65	All	≤ 25	26-45	46-65	> 65	All	
F1	70.5%	68.4%	60.2%	40.5%	60.9%	63.9%	69.5%	55.4%	37.4%	55.3%	57.9%
F2	2.8%	2.3%	7.8%	6.2%	4.9%	10.5%	8.5%	4.9%	5.4%	6.5%	5.8%
F3	6.1%	13.6%	9.8%	12.5%	11.1%	15.5%	12.3%	13.5%	14.2%	13.5%	12.4%
F4	2.3%	1.5%	1.7%	2.1%	1.8%	3.1%	1.4%	2.1%	1.4%	1.8%	1.8%
F5	18.2%	14.2%	20.5%	38.7%	21.4%	7.1%	8.4%	24.1%	41.5%	22.8%	22.1%

**Figure 2:**
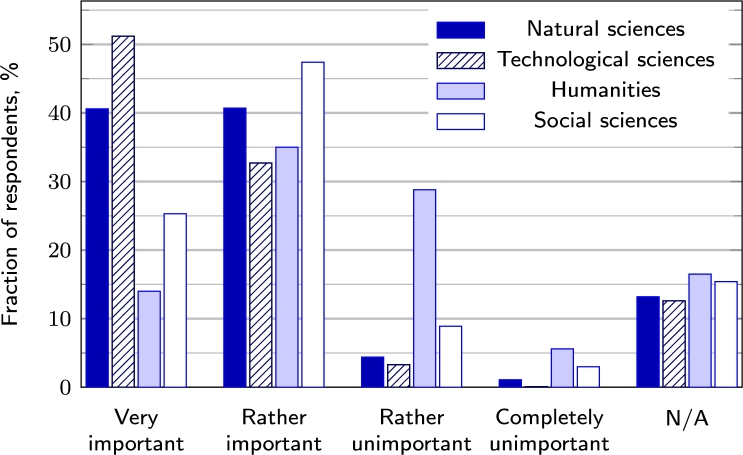
Importance of a specific science area in the education of a CS specialist according to the public opinion.

In the Omnibus survey, respondents were asked to specify the most critical science fields contributing to the education and training of CS specialists. Over 80% of the respondents selected technological and natural sciences (see Fig. 2).

Moreover, the respondents identified social sciences as contributing to the development of the CS field but with less importance. The results indicate a common understanding that education in STEM subjects is a primary path into the CS field. On the other hand, the findings highlight the necessity to inform public society (particularly the younger generation) about less technologically focused CS roles, e.g. legal advisor, data protection officer, or physical security penetration tester.

### 4.2 Survey of executive officers

The survey of top managers in private and public organisations, including human resources managers, focused on the following four questions:

(a) importance of CS to their organisation;
(b) attitude towards the tasks of CS specialists;
(c) opinion about the balance of hard vs soft skills of a CS specialist;
(d) future demand for CS competences.

In total, 64% of the executives stated that cybersecurity is very important for their business. An organisation's size was directly related to the expressed importance of CS (see Table 3). An averaged value of the CS importance on a scale from 1 (most important) to 5 (least important) grows uniformly with the yearly turnover of surveyed companies. A relatively low turnover and fewer employees in smaller organisations limit the resources allocated for CS. Such attitude of CEOs further increases the vulnerability of SMEs.

**Table 3:** CS importance (on a scale 1 to 5 where 1 is the most important) depending on the company size.

Turnover (M)	<0.145	0.145-1	1-5	5-10	>10
Number of valid responses	13	36	69	36	34
CS importance	3.308	2.583	2.290	2.250	1.941

The majority of CEOs (81% in total, whereas 84% in the public sector) expressed an opinion that an IT specialist could either fully or at least partially carry out the tasks of a CS specialist. In contrast, only 13% of the respondents think that dedicated CS roles should take the tasks. In general, 51% of surveyed companies have neither a CS nor an IT specialist. It is not surprising that a company assigns all IT-related functions, including all CS-related responsibilities, to an IT specialist whenever one can be afforded. Only the largest companies have enough resources for a dedicated CS specialist (or specialists).

**Figure 3:**
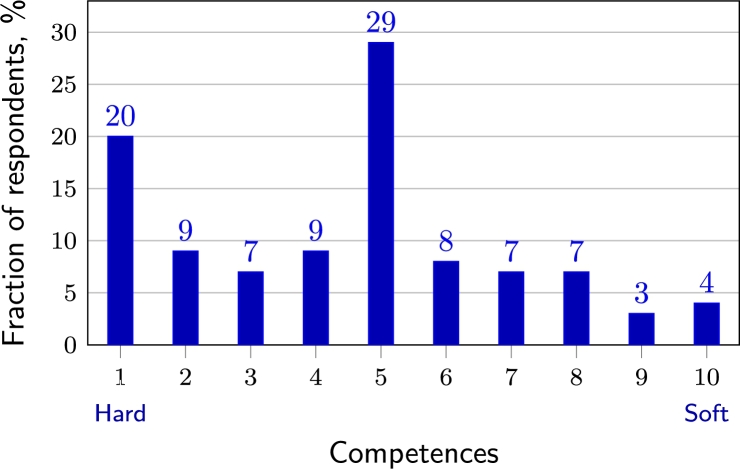
Expected balance of hard vs soft skills of a CS specialist according to top executives.

When asked to rate the priority of hard vs soft competences of a CS specialist on a scale of 10, where 1 denotes purely technical (hard), and 10—purely soft skills, the CEOs indicated (see Fig. 3) that technical knowledge is more important. The average value of the responses was 4.3 (where the middle point between the hard and soft skills was 5.5). This result was independent of the size of the company or the number of its CS specialists. However, the results differed depending on the sector: the public sector average was 3.6, and the private sector—4.6, indicating a stronger preference for technical skills in the public sector (and possibly a more apparent separation of roles).

**Figure 4:**
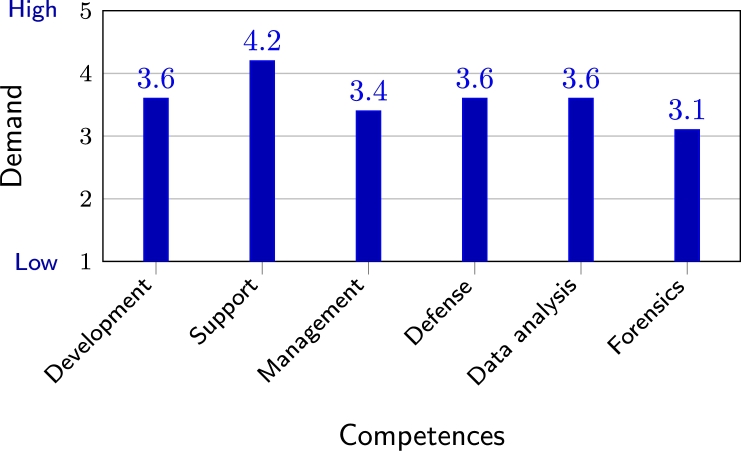
Future demand for competences in CS sub-domains (scale 1 to 5) according to top executives.

The executives were also asked to rate the future demand (next 2–5 years) for CS competences in different field domains. Even though all areas received high grades (see Fig. 4), the least attention was paid to digital forensics, the systems support was deemed the most important. Thus, continuous business operations are prioritised. At the same time, managers expect to avoid CS incidents. The findings further explain their dominating opinion that an IT specialist (the one who takes care of continuous operations) should be able to take care of cybersecurity (and therefore avoid incidents).

### 4.3 CS expert survey

The exploratory study focused on the following elements to explore the perspective of CS specialists:

(a) opinion about the balance of hard vs soft skills of a CS specialist;
(b) demand for CS specialists in different CS areas;
(c) CS roles present in organisations.

The specialists put more emphasis on hard competences when asked about the balance of skills a CS specialist should have. The average score was 3.6 (where 1.0 would mean purely technical skills), substantially lower than the answer given by top executives (their average score was 4.3). Thus, managers expect the CS specialists to have more soft (communication, reporting) skills than the specialists themselves. In general, the managers seem to have a lot of expectations from their technical staff. According to them, a usual IT specialist should handle all IT areas, take care of their security, and simultaneously have sufficient communication (and possibly managerial) skills.

In an open-ended question, the experts were asked to indicate the most in-demand CS specialist roles. The highest-scoring answers were: security architect, forensic specialist, and penetration tester. The experts mentioned technical roles twice as often as managerial CS roles. Judging by the occasional mention of purely IT roles (network administrator or operational technology specialist), even among CS experts, cybersecurity is often not separated from IT. Interestingly, no one has identified any education, research, or risk management roles.

One of our aims was to find the distribution of CS roles in Lithuania organisations. We provided ENISA ECSF and NIST NICE roles to the experts. ENISA ECSF roles had ten choices because we merged the roles of Educator and Researcher, and Architect with the Implementer also made a single choice. We also selected 41 of NIST NICE roles (a few unlikely ones related to offensive operations were eliminated). The respondents had to indicate those that were present in their organisation.

On average, 67% of ENISA roles (6.7 out of 10) and 57% of NICE roles (23.2 out of 41) were marked by the respondents. When considering the ENISA framework, the CISO role is the most frequently chosen (90% of responses), followed by the Data Protection Officer and the Auditor (83% each), and the least chosen are the Digital Forensics Investigator and the Educator/Researcher (59% each). It has been observed that the number of roles increases depending on the size of the organisation, but even in very small companies (where there is only one person in the CS role), the number of CS roles remains very high. As a result, most organisations have people working in multiple CS roles.

Similar conclusions are reached if the NICE framework is used instead. The only difference was that in this case, the roles marked most often were classified as IT roles rather than CS roles according to the European classification. The role of System Administrator was selected 90% of the time, followed by three other roles: Incident Resolver (a role also included in the ENISA classification), Technical Support Specialist and Network Operations Specialist, each with 86% of the votes. The roles of Cyber Intelligence (21%) and Cyber Lawyer (26%) were mentioned the least often, as the role of the Lawyer is separate from the role of Data Protection Officer in this classification. To summarise, all CS roles are relevant in Lithuania organisations, whatever the classification, but even such a small sample shows a reduced focus on cyber threat intelligence/hunting, research (and potentially education), and cyber law.

### 4.4 Investigation of job postings

Job postings are a tool for attracting new employees, easily accessible to both small and large companies. Employers usually include detailed requirements for candidates and detailed job descriptions in their postings. We analysed the collected data not only qualitatively but also quantitatively. This analysis has provided a fairly accurate cross-section of the cyber and information security labour market.

According to Eurostat, in 2021, ICT workers in Lithuania represented around 4% of the total workforce [12]. The National Statistics Department reports similar numbers: in the first quarter of 2022, more than 3% of the employed population worked in the ICT sector. In 2019, 8.6% of companies were hiring or looking for IT professionals, and as many as 58.8% of companies had job vacancies that were difficult to fill.

The job postings were collected over one year in four rounds (see Table 4). In total, 4023 adverts were inspected in the *“Information Technology”* category on one of the largest national CV portals and the international professional social network LinkedIn. In the LinkedIn search, the results were filtered by the country. Of the total number of ICT job postings (4023), 171 were attributable to cyber and/or information security specialist positions.

**Table 4:** Number of analysed job postings.

Date (d/m/y)	Source	ICT job postings	CS job postings	CS fraction
28/03/2021	National CV portal	936	14	1.5%
05/03/2021	LinkedIn	126	23	18.3%
01/07/2021	National CV portal	908	21	2.3%
01/07/2021	LinkedIn	181	23	12.7%
10/04/2022	National CV portal	1072	42	3.9%
10/04/2022	Direct advertisements	n/a	4	n/a
10/04/2022	LinkedIn	800	48	6.0%

Total:		4023	175	Average: 7.5%

It should be noted that the demand for cyber security professionals varies from one date to another. These changes may be related to the COVID-19 pandemic and its impact on the labour market. For this reason, 94 job posting samples collected on April 10, 2022, were chosen for the detailed analysis. To this date, most of the COVID-19-related restrictions had already been lifted in most European Union countries.

Around 5% of ICT vacancies were in cyber and information security. In the second quarter of 2022, around one in 20 new ICT professionals was expected to start working as a cybersecurity specialist or manager.

ICT (33% of job advertisements) and finance (36%) companies were the most likely to be looking for cybersecurity professionals. There were also shortages in the financial management, insurance, information provision (public sector), transport, pharmaceuticals, auditing, energy, marketing, and manufacturing sectors (30%). It should be noted that in almost 10% of the job postings, companies were looking for a specialist to provide cyber or information security consultancy to external clients.

The title of the posting often indicates the level of the future position, for example, “junior,” “mid,” and “senior.” The latter category usually includes managers (*lead* or *manager*). The level of the job position can also be identified by the salary offered, requirements for experience, and qualifications.

**Figure 5:**
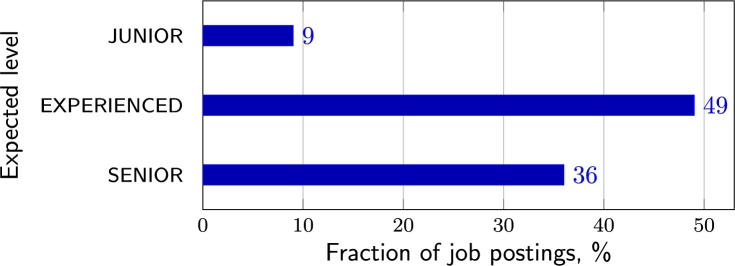
Expected experience level.

**Figure 6:**
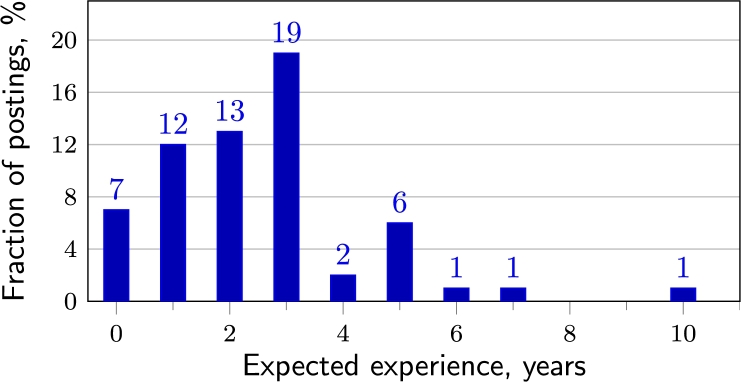
Expected years of experience.

Job postings are dominated by experienced and senior cyber and information security professionals (see Fig. 5). Entry-level positions accounted for 9.6% of all job offers only. Most companies are looking for experienced professionals (one or more years of practical experience in cyber/information security, sometimes in IT, see Fig. 6). Only 7 out of 94 job postings did not require applicants to have practical experience, and they were mostly offering an entry-level position. Sometimes experience is not defined in specific years but by adjectives such as *“extensive”* or *“deep”* (strong knowledge, excellent understanding). Many postings specify not only minimum experience but also a range of experience, for example, *“3–5 years”* or use a *“+”* sign (such as *“3+”*) next to the number of years. The experience and level requirements for applicants in the postings suggest that companies are most likely to employ experienced professionals who have worked in the CS field for several years.

**Figure 7:**
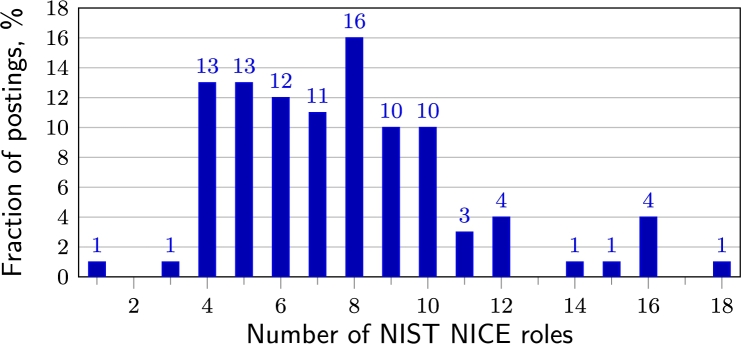
Number of NIST NICE roles in one job posting.

Linking the job roles described in the postings to the NIST NICE competence framework shows that an average cyber/information security job applicant is expected to cover 7.8 NICE roles out of 41 possible (see Fig. 7). Only one employer looked for employees with a narrow specialisation (one specific NICE role per posting). There were also postings with 16 or even 18 mentioned roles in the description.

The number of roles for beginners is usually lower than for experienced or senior positions. There is also a trend that the larger the private company or the more employees it is looking for at any time, the narrower the specialisation of the cybersecurity jobs. This trend is particularly evident when a company has a separate cyber or information security department.

The analysis shows that the NIST NICE framework is too detailed for the organisations of a small country. The ENISA ECSF framework has, therefore, just 12 roles. However, based on the job postings analysis, companies would like to reduce the list of CS specialisations by combining several ENISA roles. On average, 3.1 ENISA roles per cyber security specialist are mentioned in job postings. 77% of the job postings required the job seeker to perform the functions of two to four ENISA roles.

The popularity of ENISA roles correlates with NICE NIST roles. The most sought-after ENISA role is the *Chief Information Security Officer (CISO)*. Functions of this position were mentioned in 56% of all job postings. CISO was followed in order of popularity by *Risk Assessors and Managers* (46%), *Developers of Secure Technological and Software systems* (45%), *Auditors of Organisational Security and Procedures/Processes* (44%), and Incident Managers (41%). Meanwhile, functions of legal regulation, formal data protection, and operational GDPR enforcement professionals were the least needed (2% of all postings).

There is an interesting distribution of ENISA roles in frequency in the secondary job function descriptions. Recruits are most often expected to train colleagues (26%), assess/manage risks (19%), or investigate incidents (17%) in addition to their main job tasks.

### 4.5 Expert and focus group interviews

Results of interviews of selected experts from CS field indicate that, due to the overlap of IT and CS profiles, the career route into CS begins by acquiring IT education or experience (the essential requirements for a CS specialist are highlighted in Table 5). Basic IT knowledge, ability to read software source code, ability to program, script, use a command line and develop tools, and knowledge of network administration and cloud and virtualisation technologies, are considered a common basis for both IT and CS profiles. If an IT specialist understands CS threats and applies methods to prevent them, then he/she is considered competent by default in solving CS issues. Moreover, results demonstrate that in recruitment procedures, a higher education degree is not an essential requirement despite the recurring opinion of the experts that general IT studies provide a necessary “spectrum of knowledge” and basic competences. The main selection criteria refer to qualities acquired behind formal education, namely, experience and certificates. According to the experts, certificates indicate personal motivation and a set of abilities.

**Table 5:** The path of a CS specialist into the area (technological profile).

**IT profile**	⇒	**CS specialist**
• IT education and/or work experience		• Understanding CS threats
		• Application of CS threat prevention methods
**Skill set**	⇒	⇑
• IT Basics		**Filter**
• Reading software code		• Diploma and experience
• Software development / scripting		• Certificates
• Development of tools		
⇑ ⇑ ⇑		

**Personal features**		
• Willingness, motivation		
• Curiosity, spending extra time		
• Practice, experimentation		

Most experts recognised that a career path in the CS area could start as a system or network administrator. The initial position could be in the incident response (IR) team and performing analyst functions. A basic set of IT knowledge or experience in the IR team would lead to a further career step, e.g. implementing and managing security systems (Security engineer) or choosing tasks of less technical work, e.g. information security management, consulting, auditing, GDPR conformity assessment. CS tasks of non-technical nature (ensuring regulatory compliance, taking care of procedures or processes) require abilities to ask for information and translate technical language into business language for cross-disciplinary coordination and communication between teams. Nevertheless, these non-technical positions also require a basic level of IT knowledge to address the technical staff.

When asked to identify the roles of CS specialists in large companies, the experts named two groups of specialists: technical and non-technical. Non-technical roles cover data security officers, information security managers, regulatory compliance officers and auditors. Although compliance is separated from management in the international classifications, the overlap of roles is specific to Lithuania. According to experts, the demand for non-technical roles and specialists has increased and is still high due to the EU GDPR directive. Alternatively, the roles responsible for incident management, security implementation and day-to-day assurance (DevOps), vulnerability management and testing, and system monitoring were assigned to the technical role group. Unexpectedly, any of the experts failed to mention roles related to research & development, innovation, threat intelligence, or digital crime investigation. Also, risk management was not classified as a function of a separate role. When asked about the continuing education and certification of CS specialists, the experts emphasised the individual responsibility.

## 5 Discussion

The surveys, interviews, and analysis of job postings give several consistent results. First of all, the role of a CS specialist is deeply associated with the role of a generic IT specialist. This trend is evident in the opinion of the general population. Most CEOs also think that an IT specialist may carry out the functions of a CS specialist. Secondly, most Lithuania companies are too small to have a set of separate CS roles. However, the activities needed in the CS area are similar regardless of the company size. As a result, a usual CS specialist in Lithuania is expected to perform functions of several ENISA ECSF or NIST NICE roles, confirming our hypothesis.

**Figure 8:**
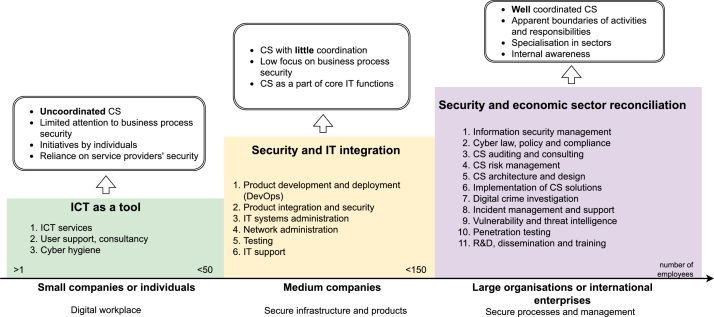
Cybersecurity implementation level versus the organisation size.

To summarise the observations, Fig. 8 maps the cybersecurity landscape in different organisations depending on their size. *Small companies or individuals* (typically with less than 50 employees) whose primary activity is not related to providing IT products or services tend to delegate CS roles to an ICT specialist within the organisation if they have one or rely on their service providers. CS is based on good ICT literacy and secure use of IT resources and data. CS functions are uncoordinated.

*Medium enterprises* (around 50–150 employees) whose primary activity is not related to the ICT sector, including small IT service provider companies, delegate CS tasks to their in-house software developers and implementers. CS is part of an organisation's core IT functions and is sometimes taken for granted as a responsibility of developers or administrators, for example, by using attractive names such as (Cyber-)DevOps–(Cybersecurity) Development and Operations. There is poor coordination of CS governance and regulation and limited coverage of incident response or other CS activities.

*Large or international enterprises*, including state-owned organisations, use more detailed job descriptions. Work activities are usually linked in a hierarchy that has explicit CS activities and is well coordinated.

**Figure 9:**
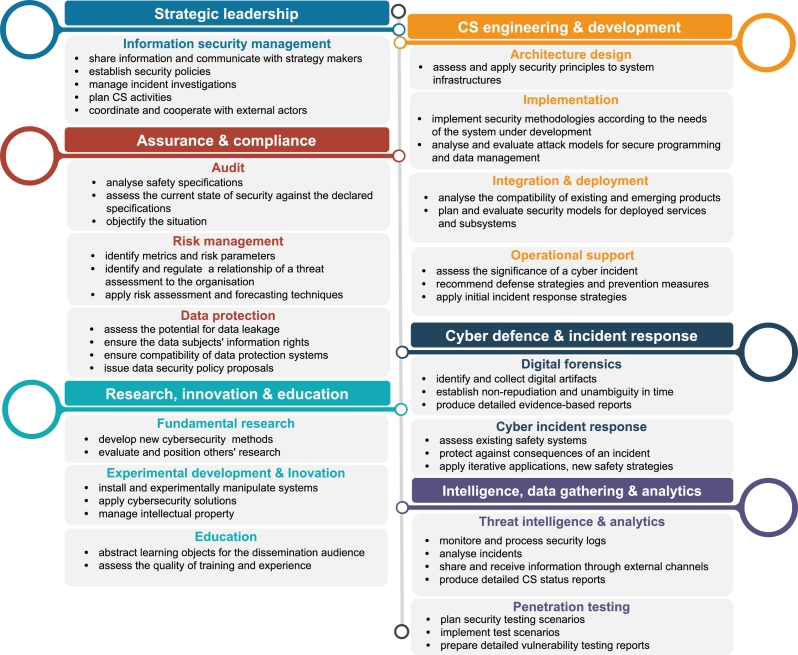
Proposed CS competence framework of a small-nation state.

Therefore, based on the research results, we propose a professional cybersecurity framework comprising six activity areas (see Fig. 9). The proposed framework is built based on cyber activities, tasks, and responsibilities within a context of a small nation state.

*Strategic leadership.* Involving senior management in mitigating cyber risks is a key success factor [6]. Establishing government procedures, continuous performance monitoring, employee motivation, and other tools developed by executives build sustainable cybersecurity awareness. Therefore, this activity area is related to strategic management in cybersecurity. Ryttare [32] performed several semi-structured interviews with respondents from six organisations in different industry sectors to identify predominant factors in establishing a security culture. For example, one of the factors is the need for a leader interested in cybersecurity with a pedagogical approach to support employees in the continuous change towards security-oriented culture. Dhillon [9] proposed three levels of organisational competence to harness IT, aiming to gain competitive advantage—strategic, exploitation, and supply. Information security must be developed in all three areas within the organisation [10]. For example, the strategic level includes competence to clearly define roles (threats are mitigated before they become serious), and the exploitation level includes competence to lead and influence others' awareness (will to “sell an action plan”). Therefore, trustworthy managers in the CISO role with its responsibilities build credibility and capacity within the organisation [22].

*Assurance & compliance.* Digitalisation processes have enabled the development of legal regulation in cybersecurity to ensure data protection and support resilience against cybercrime. Numerous policies and regulatory measures have been adopted [15] to protect fundamental rights and cover cyber issues independently of the rights. The technical multi-layered ecosystems raise challenges regarding security and safety levels [7]; for example, the European Union still lacks some regulations and mandatory requirements for the manufacturer regarding IoT product security. Solutions and strategies might lead to risks, and organisations need to analyse not only their own risk but also the industry-wide trends [23]. Therefore, the activities of this area support legal compliance, audit, and risk management.

*Research, innovation, and education.* Mushtaq [27] performed semi-structured interviews with experts to define the technology course role at secondary schools. Experts emphasised that it is more important to build secure habits than to produce citizens for the cybersecurity job. After reviewing the literature, Mwim and Mtsweni [28] concluded that cybersecurity training and education is the top cybersecurity culture factor. Educational institutions focus on education and research as a primary function, but innovation can be fostered only through cross-sector partnerships, including business and academia [34]. Therefore, the area combines education in a broad sense (including internal training) and research (academia and industry) to define the national direction toward innovation development.

*CS engineering & development.* Today, implementing security tools in compliance with standards is a “basic hygiene” [33]. Therefore, this area considers any tool application and solution integration as an implementation in the infrastructure or software, starting from the architecture design to the operational support. The activity scope of this area is IT system security and all objects and entities interacting with these systems in the cybersecurity context as proposed by Villalón-Fonseca [39]. It is important to emphasise that engineering and development are not limited only to IT systems but also cover industrial control systems. Of course, for example, SCADA security and IT security differences like communication protocols and fault tolerance levels should be considered in the engineering processes [38].

*Cyber defence & incident response.* Incident response can be seen as a separate group of activities and responsibilities. The extensive availability of services via online systems and usage of smart devices pre-program possible attacks and compromises. Reporting processes to national CSIRTs are predefined by legislation, but some enterprises are required to maintain incident response procedures and test them for preparedness. Information officers are responsible for ensuring procedure compliance and training, while incident management involves teamwork. This activity's scope covers cyber defence from the incident response perspective, including digital forensics, which is a part of the incident response process [17].

*Data gathering, intelligence & analytics.* This activity area combines analytics of the data and testing system vulnerabilities. Monitoring tools generate extensive amounts of data, and the question of how much data are translated into the decision remains even today [33]. Therefore, proper data analysis provides the system view of daily status to compare to exceptional situations, unexpected behaviour, and publicly shared indicators of compromise. Organisations can benefit from sharing information and using the available threat information. Intelligence-sharing communities are a powerful tool to get the solution and avoid the risk of being targeted via vulnerabilities [25]. Large data amounts require technological solutions to analyse and detect attack patterns or irregularities within systems. Therefore, the activity area relates to challenges and needs for real-time identification of vulnerabilities, for example, by applying artificial intelligence [26].

## 6 Conclusions and future work

Our investigation's primary goal was to test and evaluate the application of currently well-known CS competence frameworks in a small nation state. We designed a comprehensive research methodology workflow, cross-examined the CS field in Lithuania using several data collection methods and an inductive approach, and proposed a new competence framework. We found that existing CS competence frameworks do not apply to the majority of organisations, hence the need for the activity-based generic framework. Our discussed findings directly link the national efforts to compete in the global cybersecurity job market.

The proposed framework for the CS field applies to organisations for defining consistent job descriptions, communicating educational paths, and identifying key-stone issues related to developing organisational strategy regarding CS. The framework presents a balanced view of the cyber workforce categories and reflects the existing international standards.

Focus on a small state creates limitations of the proposed framework. The framework's hierarchical structure balances workforce proportions found during the research. But small countries are sensitive to changes in local innovation and investment intensity, and a new big player can significantly impact workforce distribution. The business changes might prioritise some CS competences over others and shift the specialist profiles into specialisations at the country's level. However, we cannot say that the framework does not suit large countries, and further investigation is needed. Finally, the industry should validate and approve the framework for educational and professional purposes.

As a future research direction, we envision other detailed analyses of data gathered during surveys and interviews. It is important to explore the gender balance in CS and how to promote women in the labour market. Continuing our research, we also intend to investigate possible options regarding early and late exposure to cybersecurity competences in education.

## Declaration of Competing Interest

The authors declare no competing interests.

## Data Availability

Open access data as aggregates has been released for further research and reference:1. Survey of general population, aggregated data [21]; 2. Survey of CEO and human resources management aggregated data [2]; 3. Analysis results of job postings, aggregated data [3].
